# Title: Immunotherapy; a ground-breaking remedy for spinal cord injury with stumbling blocks: An overview

**DOI:** 10.3389/fphar.2023.1110008

**Published:** 2023-01-25

**Authors:** Yasmeen Saeed

**Affiliations:** Provincial Key Laboratory for Utilization and Conservation of Food and Medicinal Resources in Northern Guangdong, 288 University Ave. Zhenjiang District, Shaoguan City, Guangdong Province, China

**Keywords:** spinal cord injury, myelin associated inhibitors, DNA vaccination, immunotherapy, immunoregulation

## Abstract

Spinal cord injury (SCI) is a debilitating disorder with no known standard and effective treatment. Despite its ability to exacerbate SCI sequel by accelerating auto-reactive immune cells, an immune response is also considered essential to the healing process. Therefore, immunotherapeutic strategies targeting spinal cord injuries may benefit from the dual nature of immune responses. An increasing body of research suggests that immunization against myelin inhibitors can promote axon remyelination after SCI. However, despite advancements in our understanding of neuroimmune responses, immunoregulation-based therapeutic strategies have yet to receive widespread acceptance. Therefore, it is a prerequisite to enhance the understanding of immune regulation to ensure the safety and efficacy of immunotherapeutic treatments. The objective of the present study was to provide an overview of previous studies regarding the advantages and limitations of immunotherapeutic strategies for functional recovery after spinal cord injury, especially in light of limiting factors related to DNA and cell-based vaccination strategies by providing a novel prospect to lay the foundation for future studies that will help devise a safe and effective treatment for spinal cord injury.

## 1 Introduction

Spinal cord injury (SCI) is a devastating disorder caused by the loss of the nerve connection between the cerebral cortex and the peripheral nervous system, resulting in permanent or temporary modifications to the nervous system’s motor, sensory, and autonomic function, while imposing negative impacts on respiratory, bowel and urinary, and sexual function (Aziz et al., 2014). Non-etheless, a growing number of scientific studies have also documented the prevalence of neurological and psychological disorders in chronic SCI patients (Huang et al., 2017; Sefiani and Geoffroy, 2021), which indicates that SCI not only leave deleterious effects on a person’s physical health but also impose mental health issues which ultimately deteriorate overall wellbeing (Li et al., 2019; Ren et al., 2020). Moreover, limited or no regenerative ability of the central nervous system (CNS) in adult mammals along with the presence of a hostile micro-environment, glial scarring, and cavitation after traumatic injury is attributed to the permanent functional, motor, and sensory loss. Given the latest understanding of the pathology of SCI, autoimmunity (a hallmark of SCI’s pathology that culminates in the accumulation of auto-reactive immune cells) has been indicated to exacerbate its detrimental effects by affecting the renal, reproductive, and cardiovascular systems (Jones, 2014).

However, to date, available treatments for SCI such as non-pharmacological (decompression, stabilization, rehabilitation, CSF drainage) and pharmacological therapies (Minocycline, ketorolac, riluzole, curcumin), are mainly focusing on drugs and surgical interventions such as surgical decompression, rehabilitation training techniques, and nursing care to promote recovery and prevent further damage (Li et al., 2019; Venkatesh K et al., 2019; Ren et al., 2020; Al Mamun et al., 2021). Yet none of these strategies has achieved any positive outcome. Another important intervention for SCI therapy is cell transplantation (MSCs, NSC), iPSCs, and cells with scaffold transplantation therapies (NeuroRegen scaffold + BMMCs) (Venkatesh K et al., 2019). However, despite gaining significant traction as a potential treatment with promising outcomes in preclinical trials for SCI over the past decade (Kumar et al., 2014; Nagoshi and Okano, 2017). Yet, clinical trial data present differing opinions with no clear conclusion due to failure of neural recovery coupled with disorganized or non-existent axon regeneration (Taveggi et al., 2010; Abbas et al.,2020). Some clinical trials have even reported adverse effects associated with these strategies (Gong et al., 2020). The other reason for the lack of an effective therapeutic strategy can be attributed to the fact that most of the research work and therapeutic intervention remained focused on the improvement of functional recovery of motor and sensory processes while giving less or no attention towards the recovery of other affected systems that deteriorates the quality of life after SCI, i.e., bowel and bladder function, muscular spasm (a very common condition found in people living with SCI), and increased risk of psychological complication, peculiarly, schizophrenia (Fehlings et al., 2018). Therefore, detailed studies investigating the root cause of these above-mentioned dysfunctions can pave the path for a breakthrough in the field of immune-based therapy for SCI.

Importantly, immune cells are known to possess an arbitrary role in degenerative processes following SCI, however, their ability to adopt regulatory and pro-regenerative phenotype by modulating their micro-environment makes inflammation a unique process for injury and repair dichotomy to control tissue injury (Popovich and Longbrake, 2008; Alizadeh et al., 2018). This correlation between neuroinflammation and wound healing holds the potential to bring positive functional outcomes after SCI which ultimately leads to the concept of immunotherapy (Karp and Leng Teo, 2009; Kwiecien et al., 2020). Encouraging outcomes of research studies focusing on inflammatory response during secondary damage have suggested that precise identification of its unique signalling pathways (involved in inhibition or maintenance of myelination and promoting the remyelination (a key factor in neuronal recovery)), can aid in developing an efficient neuroprotective and regenerative modality that could not only replace the damaged cells but also promote the restoration of axonal growth and neuronal communication in the spinal cord (Karsy and Hawryluk, 2019; Shao et al, 2019). For instance, the root cause of muscular spasms in spinal cord injury is attributed to the demyelination of the axon that deteriorates the normal function of spinal circuitry (Li et al., 2020). Intriguingly, preclinical evidence from SCI-rats models has shown that anti-Nogo antibodies immunization promotes remyelination and reduced muscular spasms (Li et al., 2020; Sefiani and Geoffroy, 2021). Thus the significant role of immune response in CNS recovery and darn lack of effective therapeutic modality indicates the importance of immunotherapy-based treatment for CNS disorders. For instance, immunotherapeutic strategies have shown positive outcomes for the treatment of many CNS degenerative disorders, such as Alzheimer’s disease (AD), Parkinson’s disease (PD), stroke, and multiple sclerosis (MS) (Hussain et al, 2018). Yet, the clinical implication of immune-based therapy for SCI lacks the standard to become a universally accepted therapy (Dekaban and Thawer, 2009). Therefore, understanding the cardinal concept of immunoregulation and its main regulatory events can help in laying down the foundation of an effective immunotherapeutic strategy for spinal cord injury (Dekaban and Thawer, 2009). However, it is important to ensure the efficacy and safety of these strategies by overcoming the lack of understanding about key molecular events and their associated signalling network. Accordingly, this study aims to provide an overview of previously reported studies focusing on immunotherapeutic strategies, and their limitations in the context of cellular or DNA vaccination strategy, and to provide a novel perspective for establishing an efficient therapeutic approach in future.

## 2 Cross-talk between neuronal recovery and capacity of neuroinflammation to induce myelination after SCI

Though the immune system has been known to aggravate sterile inflammatory responses after CNS injury. However, under healthy physiological conditions, the immune system holds a significant role in tissue homeostasis (Putatunda et al., 2020; Negi and Das, 2017). For instance, the immune system possesses enough potential to not only protect the body’s tissue from damage but also direct the recovery of damaged tissues (Zhou *p* et al., 2019). Therefore, a comprehensive understanding of immune response and associated pathophysiological mechanisms through which major immune cells i.e., microglia and astrocytes in the central nervous system (CNS) modulate the neuroinflammatory responses and regulate their immunophenotype to suppress robust inflammation after SCI can not only facilitate injury prognosis but also provide a key to formulate an effective therapeutic strategy (Pang QM et al., 2022). Classically, SCI consists of acute and chronic phases, whereas due to its complex pathophysiological mechanism, an acute injury is further categorized into primary and secondary phases (Sefiani and Geoffroy, 2021). The primary phase occurs right after the onset of traumatic incidence of acute injury and tends to cause irreversible damage (Huang et al., 2017; Alizadeh et al., 2018). The intensity of this phase actuates the outcome and severity of SCI (Yoshizaki et al., 2019). Alternatively, the secondary phase of injury describes the series of cellular, molecular, and biochemical processes that continue to self-destruct spinal cord tissue and ultimately halt neurological recovery (Alizadeh et al., 2019). Peculiarly, the inflammatory response from secondary injuries is considered a prime catalyst and the most crucial phase in the pathophysiology of SCI (Cofano et al., 2019). On the other hand, it is also important to mention that inflammatory response to injury also plays an important role in regulating axon regeneration after spinal cord injury. Accordingly, intriguing research has suggested that an anti-inflammatory response can potentially break down the early inflammatory response and aid in rehabilitating the damaged tissue to a normal state (Kaur C et al., 2017). For instance, it has been reported that M2 macrophage transplantation can not only provide neuroprotection but also aid in the regeneration of neuronal cells in various animal models (Papa S et al., 2016). More importantly, this neuroprotective and neurodegenerative response of macrophages transplantation has been reported to be attributed to the reduction of myelin-related glycoprotein, which ultimately promotes axon regeneration and myelination (Miron VE et al., 2013; Zhou *p* et al., 2019). Hence, these studies speculate that modulation and regulation of inflammatory response in the secondary phase of SCI could be an indispensable target for establishing an effective therapeutic intervention that can aid in the reconstruction of neural circuits by promoting the axon and myelin formation (Tran et al., 2018).

Further insight into the pathophysiological mechanism reveals that SCI disrupts vascular supply to the spinal cord causing hypoperfusion which results in hypovolemia and hemodynamic shock due to excessive bleeding and neurogenic shock (Mohammed et al., 2020). While overflow of blood in ischemic tissue produces free radicals and triggers an inflammatory response that leads to ionic imbalance, excitotoxicity, and oxidative damage (Wang et al., 2015) causing extensive deterioration of all cells and vascular components that succumb the spinal cord to necrosis and haemorrhage. These extensive necrotic lesions and neighbouring surviving tissues are considered a major contributing factor in the formation of a cavity of injury (COI) within the spinal cord, which represents an area of inflammation and fluid accumulation (Kwiecien et al., 2020). Further studies have suggested that during the second phase of injury inflammatory granulomatous cells such as fibroblasts, capillary blood vessels, and macrophages invade the necrotic area from the subarachnoid space and resulting in fibrosis of arachnoiditis, forming a typical scar surrounded by an astroglial reaction (Kwiecien et al., 2020). Therefore, it is hypothesized that the inhibition and elimination of inflammation during the second phase *may aid in* combating a spinal cord anti-inflammatory activity, specifically astrogliosis (Kwiecien et al., 2020). Additionally, astrocyte-specific inflammatory signalling is the main factor in deteriorating secondary injury after SCI (Ahmed et al., 2018). These reactive astrocytes produce axonal growth inhibitors that prevent axonal regeneration and ultimately result in glial scar formation (Okada et al., 2018). The presence of glial scar or CNS gliosis is considered a major factor that halts the process of regeneration in CNS and makes it more complicated and difficult as compared to the PNS (because the small lesion in PNS damages can potentially regenerate) (Momenzadeh and Jami, 2021). Demyelination and axon regeneration inhibition is a central event of SCI pathophysiology in which myelin cells and oligodendrocytes play an important role. Since myelin is a lipid-rich nerve sheath that is comprised of glial cell’s plasma lemma layers and possesses a key role in signal conduction while myelinating cells possess the potential to induce self-survival signals to alter the extracellular environment in favour of myelination (Jessen and Mirsky, 1999). These signals also affect axon development or repair, the outgrowth of the detached axon that ultimately supports and reconstructs damaged fibre tissue. Moreover, the formation of glial scar cystic cavities and producing glial scar, myelin inhibitor, and inflammation are among the major challenges that negatively impact the repair of the spinal cord. Although at early stages of spinal cord injury, glial scar prevents the spread of damage to neighbouring healthy tissue to protect the delicate surrounding tissue (Faulkner JR et al., 2004; Zhou *p* et al., 2019). However, the advancement of damaged glial scars creates a physical and chemical barrier which prevents the migration of myelin restorative progenitor cells to the site of injury (Momenzadeh and Jami, 2021). This suggests that SCI not only deteriorates the axon-myelin contact (that is essential for the development and regenerative signals of myelination) (Ortiz et al., 2016) but also disrupts the axon-glia network that results in the activation of astrocyte’s products such as fibronectin and chondroitin sulfate proteoglycans (CSPG) and myelination inhibitors. Ultimately, altering the extracellular matrix texture in gliosis and causing the demyelination of axons (Huang et al., 2019). Intriguingly, chondroitin sulfate proteoglycans have been indicated to be significantly downregulated by the inhibition of astroglial NFκB (Brambilla et al., 2005), therefore, chondroitin sulfate proteoglycans modulation can be an effective strategy to improve functional recovery and neuroregeneration after CNS injury (Rauvala et al., 2017).

More importantly, in CNS, myelin-associated inhibitors (MAIs) have been indicated to reduce the re-growth and development of neurites into peripheral nerve grafts in cultured or implanted neurons (Wang et al., 2015). For instance, MAIs, such as Nogo-A (Oertle and Schwab, 2003), MAG (Spencer 2003), and OMgp have been reported as major reasons for the failure of regeneration after CNS injury in the adult. Additionally, oligodendrocytes and astrocytes associated inhibitory molecules i. e, tenascins (TNs) and chondroitin sulphate proteoglycans (CSPGs) (a product of reactive astrocytes or oligodendroglia) could potentially result in the formation of glial scar and form a physical barrier against axonal regeneration after injury (Jones et al., 2003; Silver and Miller, 2004). Therefore, immunotherapy targeted on the myelin inhibitors and their receptors (as well as neutralizing agents, such as antagonists, and antibodies) is considered a promising therapeutic candidate for axonal regeneration and functional recovery after spinal cord injury. Despite causing great damage to the affected area in most demyelinating conditions, the acute inflammatory response has been known to contribute to atoning the inhibitory effects of degraded myelin by activating the regenerative growth factors at the lesion site (Baer et al., 2009; Li et al., 2020). This concept suggests the vital role of the immune system in the regulation of demyelinating conditions to promote myelin regeneration (Momenzadeh and Jami, 2021). Therefore, immunization with MAIs self-antigens can be a promising immunotherapeutic strategy for the treatment of SCI.

To date, available research has shown both the effectiveness and ineffectiveness of immunotherapeutic approaches such as anti-inflammatory therapy and the DNA vaccines targeting MAIs and their related receptors in signal transduction (Wang et al., 2015; Momenzadeh and Jami, 2021). Yet, at the moment it is difficult to make any statement due to a lack of comprehensive understanding of key molecular factors and their signaling networks associated with motor and sensory dysfunctions after the incidence of SCI. Therefore, to achieve a successful outcome in terms of repair and functional recovery after SCI, it is necessary to unravel the crosstalk between the resident and invading immune cells contributing to the initial inflammatory damages and late neurorepair process. For this purpose, it is also essential to precisely identify the function and characteristics of innate and adaptive immune cells after the onset of CNS injury (Putatunda et al., 2018). Moreover, understanding the mechanisms through which self-antigens modify and co-regulate multiple targets, can also aid in SCI recovery by avoiding the potential risk of autoimmune diseases.

### 2.1 Immunotherapy targeting remyelination to aid in SCI recovery and its underlying mechanism

**FIGURE 1:**
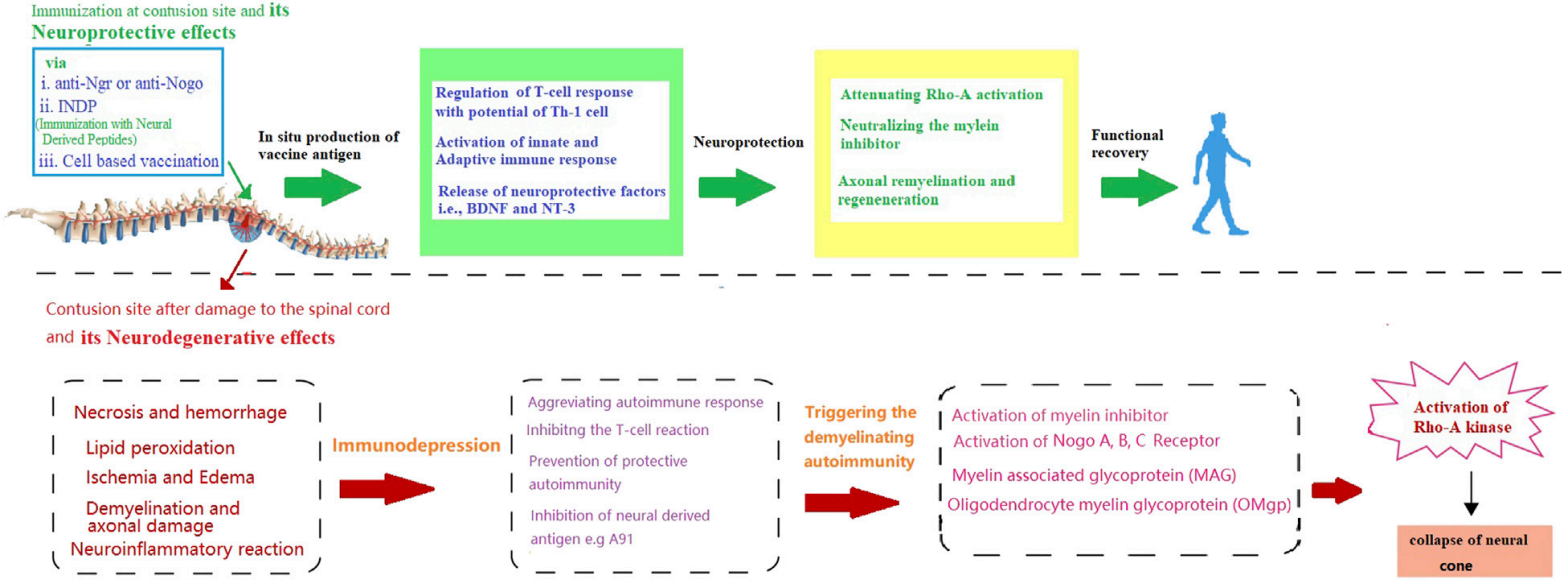
Schematic presentation of neurodegenerative effects of spinal cord injury and the possible way through immunotherapy provide neuroprotection and functional recovery.

By considering the importance of remyelination in SCI recovery, this section will attempt to unravel some key factors involved in remyelination for immunotherapeutic strategy. Although the dual nature of the immune responses coupled with the anatomical level of SCI determines the role of the immune system during injury. However, a lack of knowledge about these crucial molecular events remained the major challenge in formulating an immune-based therapeutic modality for SCI (Brommer et al., 2016; Hong et al., 2018; Ulndreaj et al., 2020). To date, it is widely known that after the onset of the injury both innate and adaptive immune responses get triggered and attack the neural tissue, which further deteriorates the damage caused by the original injury (Donnelly and Popovich, 2008). On the other hand, immune responses are also involved in the recovery of impaired neurological damage caused by SCI (Cardenas et al., 2004). Initially, the concept of vaccination was suggested as a strategy to protect patients with SCI from infections and counter the deleterious effects of immune response (Meisel et al., 2005). However, due to the significant role of immunomodulation in nerve and myelin regeneration, immunotherapeutic strategies also possess the potential to provoke the endogenous restoration of nerve structure and function. This concept has been further reinforced by the finding that dilapidation in the regeneration following central nervous system (CNS) injury in adults is not due to intrinsic properties of CNS neurons but due to the inhibitors (such as (MAIs), Nogo-A (MAG), and OMgp) of neurite outgrowth that accumulate around the injured site (Figure 1) (Jazayeri et al., 2015; Lu et al., 2019). Hence, remyelination is an important aspect to trigger the regenerative capacity of the axon and hallmark of immune-based therapy.

Since, myelin repair or remyelination is a typical response to demyelination that initiates promptly after the onset of injury, especially in PNS (Popovich et al., 2001). However, remyelination being a comparatively faster process than developmental myelination can overwhelm the whole system because retrieval of remyelinating cells requires the reproduction of oligodendrocytes in CNS and reprogramming of Schwann cells in PNS. Therefore, regulation and appropriate control of the remyelination process is an essential prerequisites for myelin regeneration at the lesion site (Becker, 2016). Further insight into its mechanism indicates that remyelination is attributed to the complex molecular mechanism in which glial cells possess the major role of making a favourable microenvironment at the site of the lesion for remyelination (Momenzadeh and Jami, 2021). However, the survival of glial cells depends on different factors, i.e., underlying cellular pathways and neurotrophic factors such as PI3 kinase/AKT pathway to provide trophic support and to aid in survival, proliferation, and differentiation of glial progenitor cells (Flores et al., 2000). Among MAI’s, Nogo receptors and their associated mechanism including degraded myelin components (Oligo myelin glycoprotein (OMGP), MAG, and Nogo-A), extracellular matrix inhibitors, and astrogliosis-induced substances (endothelin 1, CSpGs, Hyaluronan and fibronectin), possess the key role in the suppression of myelination inhibitors for endogenous spinal cord repair system (Maier et al., 2009; Niknam et al., 2019). Further insight into its molecular mechanism indicates the role of three main pathways, i.e., RhoA, Jagged/Notch, and WNT, in blocking neuronal cell differentiation. Intriguingly, a recent study has found that LINGO-1 (a functional component of the Nogo “neurite outgrowth inhibitor” receptor) is among the most important myelination inhibitor that possesses the potential to selectively express in oligodendrocytes and neurons (Huang et al., 2019). These protein components of Nogo can potentially suppress myelination *via* negative regulation of oligodendrocyte differentiation, inhibition of protein kinase B (Akt) phosphorylation, and activation of the RhoA pathway. Whereas, inhibition of LINGO-1 has shown positive effects on survival and remyelination of neurons and oligodendrocytes which significantly promote functional recovery in animal models (Huang et al., 2019). In addition to regeneration and functional recovery, the other positive aspect of antibodies against myelin-associated inhibitors MAIs (such as Nogo-A) indicates that these antibodies have never shown any association with increased pain or hyperalgesia (Maier et al., 2009). Therefore, improvement of functional recovery achieved *via* neutralization or inactivation of MAIs by antibodies without pain or hyperalgesia is of great importance concerning future clinical trials after CNS injury [34]. Besides, it is also noteworthy that Nogo receptors could be the major contributor to the emergence of schizophrenia and other mental disorders after SCI, due to their significant role in memory formation in the hippocampus and by specifically suppressing the signalling pathways responsible for increased fear extinction (Sefianiet al., 2021; Fehlings et al., 2018).

Immunotherapeutic strategies trigger interplay between “innate” immunity (mediated by neutrophils, microglia, or macrophages), and adaptive (mediated by B- and T-cell) immune systems. Innate and adaptive immune responses are considered essential for not only regulating neuroinflammation but also for CNS repair (Putatunda et al., 2018). For instance, innate immune cells include monocytes (which subsequently differentiate into macrophages), granulocytes (basophils, eosinophils and neutrophils), mast cells, natural killer cells, and dendritic cells. While neutrophils have been indicated to cause BBB breakdown and result in brain edema formation (Aubé et al., 2014; Semple et al., 2015). On the other hand, the damaged neuronal cells result in the activation of infiltrating dendritic cells and present antigens to T-cell, leading to an adaptive immune response (Jin and Yamashita, 2016; Gucluler et al., 2017). The T-cell responses may be specific to CNS-restricted antigens. T-cell recognize antigens through their surface-bound T-cell receptor and are classified into CD8^+^ cytotoxic T-cell and CD4^+^ helper T-cell (Th). CD8^+^ T-cell detect antigens presented by MHC class I (MHC I) molecules while CD4^+^ T-cell recognize MHC II antigens primarily presented by APCs like dendritic cells, macrophages and B-cell (Itano and Jenkins, 2003). Although the classic concept of immunotherapy indicates that the efficacy of vaccination depends on a functional adaptive response, therefore, it has been widely accepted that focusing on understanding the status of the adaptive immune response following SCI at various levels could aid in designing effective vaccination strategies for neural recovery (Ulndreaj et al., 2020). Another important aspect of immune regulation in CNS indicates that despite being immune-privilege, CNS has been reported to activate T-cell that can cross the BBB (Flügel et al., 2001; Suberviola et al., 2008) and plays a pivotal role in the protective defence system by producing the neurotrophic factors in an antigen-dependent way (Moalem et al., 2000). Thus providing evidence that elevation of the autoimmune response can protect the injured CNS (Hauben et al., 2001; Ishii et al., 2012), which can improve T-cell response against foreign invaders and local production of harmful agents, excitotoxicity, and loss of trophic support from injured CNS, and ultimately prevent the secondary degeneration (Moalem et al., 2000). Importantly, it has also been unrivalled that immunization with myelin antigens such as MAIs or passive transfer of the myelin reactive T-cell could promote functional recovery and reduce neuronal degeneration in the injured spinal cord or optic nerve (Schwartz and Kipnis, 2001).

Importantly, regulatory T-cell (Tregs, that are defined by the expression of the transcription factor FoxP3 and the high affinity IL-2 receptor subunit CD25 (Josefowicz SZ et al.,2012) have recently been indicated to possess a pro-regenerative which mitigates the pro-inflammatory response to injury due to their ability to secrete TGF-β and IL-10. Moreover, Tregs have also been indicated to migrate to the sites of demyelination and directly regulate oligodendrocyte progenitor cell, differentiation and re-myelination by secretion of cellular communication network factor 3 (Sabin and Echeverri, 2020).

Hence, T-cell mediate the protection mechanism by directing against MAIs and this property of T-cell can be used to develop a potential therapeutic strategy to rescue degenerating neurons not only by reversing the inhibition of MAIs but also by boosting the T-cell response (Schwartz and Cohen, 2000). For instance, systemic immune response mediated by T helper 1 (Th1) cells (that are specific to the abundant antigens residing in the lesion site) could significantly aid in the remedial effects of inflammation (Kottis et al., 2002; Wang et al., 2015). Thus suggesting that protective autoimmunity is another key player in remyelination and supporting the concept of vaccination strategy targeting on MAIs which contribute to the production of antibodies against MAIs or their receptors and promote the functional recovery by obscuring the inhibitory effects in the injured spinal cord (Wang et al., 2015].

Non-etheless, innate immune factors i.e., cytokines, chemokines, and inflammatory mediators (produced by astrocyte and microglia in CNS) also play a key role in the success or failure of immunotherapeutic based strategies (to trigger remyelination after SCI) (Rodgers and Miller, 2012; Brambilla et al., 2014). For instance, upon onset of injury, innate immune factors respond to the injury by triggering the movement of peripheral immune cells towards the lesion site which further activates the neighbouring cells to produce neurotrophic factors and ultimately contribute to neuronal recovery (Ambrosini et al., 2005). Particularly, in demyelinating lesions, the role of oligodendrocytes cannot be ignored, since oligodendrocyte precursors regulate myelination *via* blocking pre-myelinating stages (Momenzadeh and Jami, 2021).

On the other hand, it is also noteworthy that inhibition of MAIs by vaccination is just one aspect of the treatment. For instance, a previously reported preclinical study by Huang et al. has purposed that despite targeting inflammation and related signalling pathways that promote remyelination (Huang et al., 1999), autoimmune responses also carry the risk of exacerbating the deleterious consequences of the spinal cord or peripheral nerve injury (Filbin, 2000). Non-etheless, several strategies have been designed to circumvent the risk of harmful autoimmune response, for instance, immunization with two recombinant inhibitors, such as Nogo-66/MAG, has not only diminished the risk of autoimmune response but also exhibited the efficacy in promoting axonal regeneration in the injured spinal cord (Sicotte et al., 2003). Moreover, immunization with both the recombinant DNA and protein vaccine targeting NgR (a receptor for MAIs) has been found to promote axonal regrowth and functional recovery in spinal cord injured models with no signs of autoimmune response (Jones et al., 2003; Ishii et al., 2012). Accordingly, Yu et al. indicated that an NgR vaccination (anti-NgR antibodies) can provide neuroprotection by inhibiting NgR function and other myelin-associated signalling factor inhibitors (Yu et al., 2007) by upregulating proteins that might enhance regeneration and promote neuroprotection (Teng and Tang, 2005). Moreover, combination strategies, such as neutralizing other inhibitors, cell transplantation, and neuroprotective approaches, will probably be more effective in promoting functional recovery after SCI with no side effects (You et al., 2012). Similarly, in addition to Nogo-A and LINGO-1, tenascin-R (“TN-R″ axonal re-growth inhibitor belonging to TNs members), polyclonal antibodies have also been reported to upregulate at the lesion site of the injured spinal cord (Deckner et al., 2000; Dityatev and Schachner, 2003; Woolf and Nogo, 2003). Further studies evaluating the therapeutic potential of passive immunotherapy with specific TN-R polyclonal antibodies in acute SCI injury, have shown that local administration of TN-R antibodies at the lesion sites of the spinal cord-injured rats could potentially reduce the activity of RhoA and promote the functional recovery by promoting the axonal regrowth and functional recovery in some spinal cord-injured animals (Apostolova et al., 2006; You et al., 2012). Accordingly. Figure 1 is indicating how immunotherapy can protect against the neurodegenerative effects of SCI [Figure 1].

Hence, to diminish the potential risk of autoimmune diseases by immunotherapy such as anti-inflammatory or vaccine targeting MAIs or their receptor (Wang et al., 2015), and to promote axonal regeneration *via* blocking various inhibitory signalling pathways, a further selection of protective motifs and non-encephalitogenic sequences can ensure the safety and efficacy of a MAIs-targeted vaccine (Momenzadeh and Jami, 2021; Ulndreaj et al.2020). Additionally, remyelination, neuronal protection, anti-inflammation, and upregulation of beneficial effects of astrocytes have also been reported to play an important role in improving the repair and functional recovery after SCI. Collectively suggesting that well repair and sufficient functional recovery could be realized by the choice and modification of self-antigens, and co-regulating multiple targets for the treatment of SCI.

### 2.2 Cell-based vaccination, a theoretical platform for MSC-based cellular therapeutics: A non-non-traditional vaccine strategy for SCI

The concept of cellular vaccine employs a direct transfer of pre-pulsed or transfected host cells capable of expressing vaccine antigens (Tomchuck et al., 2012). Generally, cell-based therapeutic vaccines are categorized as dendritic cell vaccines, neo-antigen vaccines, peptide vaccines, and tumour cell vaccines (Wong et al., 2016). However, the lack of resident immune cells in CNS is considered a major obstacle to an effective response to these strategies (Hu et al., 2021). The quest to find an effective therapeutic strategy has brought the idea of combining immunotherapy with dynamic characteristics of stem cells to promote functional recovery after SCI to ensure that vaccine antigens (when produced *in-vivo*) can be processed by the immune system. Moreover, to enhance the efficiency of the immune response, adjuvants are considered essential for the adaptation of cell-based vaccines in immunologically privileged environments of CNS (Hu et al., 2021). Importantly, MSCs transplantation has been reported to facilitate anatomical and functional recovery by inhibiting the expansion of glial scar formation and preventing the inflammatory response (Pang QM et al., 2021).

Therefore, to amplify their immunotherapeutic potential and to improve the vital properties of grafted cells, it is necessary to acclimatize the injected cells to the microenvironment in lesion condition in favour of myelination and control of inflammation, while the application of growth factors can control proliferation, differentiation, and migration of grafted cells (Franklin, 2002; Jin et al., 2018). Additionally, the selection of appropriate cell lineage holds key importance, not only because each cell lineage of different origin holds different potential for myelination or proliferation but also because each differentiation stage of various cell lines exhibits a different outcome of therapy (Sim et al., 2011). Although evidence from preclinical studies has reported the use of stem cells from various resources such as embryonic stem cells (ESCs), adult neural stem cells (NSCs), oligodendrocyte precursor cells (OPCs), hematopoietic stem cells (HSCT), bone-marrow stem cells (BMSCs), Schwann Cells, olfactory ensheathing cells (OECs) and dental pulp stem-like cells (DPSCs) for myelin regeneration purposes. However, CNS progenitors, peculiarly, neural stem cells (NSCs) and oligodendrocyte progenitor cells (OPC) are considered the most suitable candidate for cell population preservation. Since long-time preservation of NSC (isolated from the subventricular zone SVZ) *in vitro* can maintain their stemness potency and ability to differentiate into different neural cells such as neurons, glia, astrocytes, Schwann cells, and oligodendrocyte progenitors (Nash et al., 2015).

Initially, in the context of immunotherapy, stem cells were implicated as a combined strategy. For instance, a preclinical study used the Nogo66 receptor (NgR) vaccine combined with neural stem cell (NSC) to treat the rat model of SCI and reported a significant functional improvement compared to the use of the NgR vaccine or NSCs alone (Xu et al., 2011). Further studies provided evidence that combined administration of T cell-based vaccines and adult neural stem cells (aNPCs) into the cerebrospinal fluid (CSF) of SCI mice promoted the functional recovery after spinal cord injury which suggests the crucial role of immune response in inscribing the aNPCs to the contusion site and regulating the migration of stem cells towards acutely injured CNS sites (Ziv et al., 2006). Additionally, cell-based vaccination also possesses the potential to activate innate immune responses against peptides in MBP (myelin-based protein), which is likely to enhance the innate immunity (mediated by neutrophils, microglia, or macrophages) by promoting the body’s self-repair capability against antigens located at the site of injury (Hauben and Schwartz, 2003). Since spinal cord injury itself could trigger self-destructive processes that result in significant damage to its function, however, immune-mediated repair and maintenance can protect against self-destructive compounds that form after a spinal cord injury (Hauben and Schwartz, 2003; Wang et al., 2013). Accordingly, a recent study has reported that antigen-presenting cells (APCs) such as dendritic cells can mediate neuroprotection by inducing a T-cell-dependent immune response. It thus opens up the possibility of applying antigen-specific DCs as an efficient therapeutic strategy that can modulate neuroinflammation by transiently inducing autoimmune responses. Further studies have provided evidence that DCs primed cells can potentially trigger local adaptive immunity (mediated by B- and T-cell) by systemic immunization against antigens located at the lesion site (Wang et al., 2013). Thus indicating dendritic cell (DC)-based vaccines as promising treatments for SCI due to their ability to combat the effects of immune cells (pivotal mediators of secondary damage) (Wang et al., 2013).

Non-etheless, recently reported studies have also demonstrated that Mesenchymal stem cells (MSCs) possess the potential for remyelination enhancement (Razavi et al., 2013; Mahmoudian-Sani et al., 2017). MSCs have been suggested as a potential candidate for cell-based vaccine therapy due to their feasibility of isolation from different parts of the body such as bone marrow, adipose tissue, umbilical cord, or placenta and their dynamic properties that not only preserve their stem cell characteristics but also possess the potential to ratify immune cell-like phenotype and plays important role in immunomodulatory effects (Razavi et al., 2013). Moreover, the immunomodulatory properties of MSCs can be used to develop immune cells by upregulating the expression of CD45, MHC-II, and genes (Roep et al., 2019). For instance, immunomodulation of MSCs (Ziv et al., 2006; Favaro et al., 2016; Almeida-Porada et al., 2020) by activating them as antigen-presenting cells (APC) can be utilized as antigen-specific therapy (Berglund et al., 2017). MSC-based vaccine approaches for SCI are currently limited to a theoretical platform, indicating that they can be programmed to express hundreds of proteins across a wide range of epitopes, to act like natural infections to undergo precise post-transnational processing (Tomchuck et al., 2012). Non-etheless, evidence from proteomics analysis of MSCs has demonstrated that inhibiting the function of MCP-1 (M2-like macrophage) in the conditioned medium can remarkably reduce the MSC’s ability to induce M2 macrophages and enhance improvement in functional recovery after SCI (Hakim et al., 2019). Immunogenic modification of MSCs is attributed to the release of multiple bioactive substances, including interleukins, extracellular vesicles, neurotrophins (including BDNF), colony-stimulating factor, and Flt-3 ligand, which have been indicated to aid in functional recovery by establishing axonal contacts on motor neurons below the spinal cord injury (Sasaki et al., 2009).

Non-etheless, It is also noteworthy that stem cell-based immunotherapeutic strategies mostly utilize its immune suppression ability, because, MSCs can adapt therapeutic effects during the rescue and repair of damaged tissues according to diverse local microenvironments (Fan et al., 2020). Moreover, the immunosuppressive role of MSCs transplantation is considered a double-edged sword because suppressing the immune response can significantly enhance the risk of tumour growth (Wong., 2011). Although gene modification can increase the immunoregulatory capacity and survival ability of MSCs, however, insertion or deletion of specific genes could significantly reduce the immunoregulatory and regenerative capacity of the genome of MSCs (Pang QM et al., 2021). Therefore, the application of stem cell vaccination requires utmost caution and close monitoring, particularly, at the sub-acute stage of injury, to prevent the loss of beneficial effects of the stem cells (Ziv et al., 2006). Hence, to improve the safety, efficacy, and outcomes of cell-based vaccination, it is important to get a comprehensive understanding of their effects on the pathophysiology and associated key molecular peculiarly inflammatory response of the body after administration of a cell-based vaccine. However, due to a darn lack of precise understanding of key molecular events and their effects on the body, the concept of a cell-based vaccination approach remained elusive. In the future, the discovery and validation of novel immunogenic epitopes can also aid in recognizing the beneficial and dynamic aspect of stem cell-based vaccination strategy as a practical clinical approach against SCI (Tomchuck et al., 2012). Evidence from preclinical studies for immunotherapy.

Since preclinical trials hold great significance for clinical translation, this section will highlight some important preclinical evidence to observe the development in the application of an immunotherapeutic strategy for spinal cord injury. Although initially immunotherapy appeared as a promising concept, however, several theoretical issues and experimental outcomes hampered its success. For instance, a study by Jones et al., in 2004 using the rat model of SCI indicated the absence of any therapeutic benefit of autoimmune vaccination in SCI rats (Jones et al., 2003). Instead, they reported that myelin-reactive T-cell impaired spontaneous functional recovery and deteriorate the injury even beyond the site of trauma. However, further investigations suggested that the main reason for these conflicting results was due to a lack of precise knowledge about the complex molecular mechanisms involved in MAIs-mediated inhibitory signalling (Jones et al., 2003). Therefore, further comprehensive studies were carried out to understand the selection and modification of self-antigens by co-regulating multiple targets to bring more effective outcomes in repairing the injured spinal cord and improving functional recovery (Jones et al., 2003). For instance, a study using a rat model showed that adaptive transfer of pro-inflammatory Th1-conditioned cells posed neuroprotective effects in mice model of SCI, which support the implication of immunotherapy in the immunomodulatory treatment of CNS injury (Ishii et al., 2012). Moreover, another study using the adult rat model has indicated that immunization with neural-derived peptides (INDPs) such as A91 in SCI could significantly reduce the number of apoptotic cell’s caspase-3 activity and TNF-concentration, which ultimately promoted the functional recovery in injured rats (Rodríguez-Barrera et al., 2013). However, it is also important to note that the neuro-damaging effects of SCI and its underlying mechanism cannot simply be managed by controlling apoptosis, rather a comprehensive understanding of the cascade of molecular events is required. For instance, in the case of protective autoimmunity, the adaptive immune cells (particularly T-cell) have been reported to trigger an auto-reactive response by the identification of self-antigen (Martiñón et al., 2016). While preclinical application of synthetic peptides (i.e., A91 derived from the myelin basic protein) proved them as a potential candidate for immunotherapy to provide neuroprotection after SCI (García et al., 2012). Yet, despite these progressive outcomes, for clinical implications, it is necessary to optimize various parameters such as the dosage, safety, and schedule of these synthetic peptides.

Intriguingly, preclinical studies have shown that immunization against myelin inhibitors not only enhances functional recovery but also alleviates the devastating symptoms of SCI, including bladder dysfunction, muscle spasms, and other SCI-associated mental ailments (Fehlings et al., 2018; Sefiani and Geoffory, 2021). Furthermore, it has also been reported that purified recombinant myelin-associated glycoprotein (MAG) and oligodendrocyte myelin glycoprotein can disintegrate axonal growth cones which result in cone-retraction and neurite outgrowth inhibition in the rat model (Kottis et al., 2002). Thus providing evidence that rats immunized with antibodies targeting myelin-associated glycoprotein could potentially improve locomotor function by preventing axonal damage. In addition to the rodent model, primates have also been studied for myelin inhibitor immunizations. Such as, the macaque model of SCI showed an improvement in the control of fine fingers after anti-Nogo antibody exposure without any sensory side effects (Fehlings et al., 2018).

Further studies investigating the implication and advantages of immunotherapy suggest that rather than treating the symptoms simply with pharmacological approaches, these therapeutic strategies work by stimulating the body’s natural remedy (its immune system) (Schwartz and Kipnis, 2001; Alizadeh et al., 2018). For instance, a study using a moderately severe compressed SCI model of Sprague-Dawley rats showed that systemic bioavailability of neuronal derived growth factor i.e., Neuregulin-1 (Nrg-1), can potentially trigger an immune response that encourages repair and regeneration in the injured spinal cord. Thus suggesting a novel therapeutic target for treating traumatic SCI and other CNS neuroinflammatory conditions (Alizadeh et al., 2018). The summary of preclinical studies has been presented in Table 1 (Table 1).

**TABLE 1:** Summary of studies using immunotherapy to treat spinal cord injury.

Sr#	SCI model and species	Type of therapeutic intervention	Time and dose administration	Efficacy and important findings of the study	References
1	Moderately severe compressive SCI contusion in female Sprague-Dawley rats	Systemic immunization of SC contusion model with Neuronal derived Neuregulin-1 (Nrg-1). Nrg-1 stimulated a regulatory phenotype in T and B-cell and augmented the population of M2 macrophages in the spinal cord and blood during the acute and chronic stages of SCI.	Nrg-1 (2 μg/day) or saline (control group) was delivered subcutaneously through osmotic mini-pumps starting 30 min after SCI to investigate its effects on spinal cord immune response as well as cytokine, chemokine, and antibody production	Systemic Nrg-1 therapy augments regulatory populations of macrophages, T-cell, and B-cell both peripherally and in the injured spinal cord tissue during the acute and chronic phases of SCI. Moreover, Nrg-1 treatment promotes pro-regenerative immune mediators such as IL-10 and CCL11 while attenuating pro-inflammatory cytokines and chemokines, IL-6, IFN-γ, CXCL1, CXCL2, and CXCL3	Alizadeh et al. (2018)
2	SC contused rats at T9 level of spinal cord	In SC-contused rats, two experiments were performed. In the first experiment, animals were immunized with a neural-derived peptide A91 and MBP, 7 days before the injury. In the second experiment, animals were tolerized against SC-protein extract by the functional elimination of CNS-specific T-cell and then subjected to an SC injury	The immunization strategy was based on the fact that immunization, with any immunogen, will provide the boosted response after 4–6 days. 30 min after intramuscular injection of a mixture of ketamine (50 mg/kg) and xylazine (10 mg/kg) and then contusion was inflicted at the T9 SC-level. Sham-operated rats immunized with PBS were also evaluated. Three days after sham operation or SC-contusion	This study suggested that boosting PA by immunizing with neural-derived peptides (A91) protected the myelinated axons, and rubrospinal neurons promote better motor recovery in SC-contused rats and provide neuroprotection by decreasing LP after SC.	Ibarra et al. (2010)
3	Adult female Sprague- Dawley (SD) rats were subjected to a dorsal hemisection contusive SCI model	Human NgR containing a leading peptide was fused with the Fc fragment derived from human IgG1. The recombinant plasmid pcDNA3.1-hNgR-Fc was identified by restriction endonuclease digestion and confirmed by DNA sequencing analysis followed by vaccination of SCI model where it stimulates the production of anti-NgR antibody to overcome NgR-mediated growth inhibition after spinal cord injury (SCI)	At 6 weeks and 8 weeks after the anti-NgR DNA vaccination, the sera from vaccinated rats were collected for antibody detection and the lysates of neuroblastoma cells that endogenously express NgR was used to react with the postvaccination sera. Fc region of human IgG1 into the DNA vaccine improved the efficiency of antigen presentation by targeting the antigen to antigen-presenting cells (APCs) because most APCs express Fc receptors	This study shows that induction of anti-NgR antibodies after immunization blocked some inhibitory activities of the myelin inhibitors and, therefore, promoted axonal regeneration in some NgR-immunized rats after SC contusion as compared to the control. However, few immunized models shows no axonal regeneration after immunization	Yu et al. (2007)
4	Adult female SD rats were inflicted with spinal cord dorsal hemisection	Passive immunization of rat SC contusion model with tenascin-R (TN-R) polyclonal antibody for this purpose fragment of peptide (CDSEYSGDDCSELRCP) was designed and synthesized according to the amino acid residues of epidermal growth factor-like domains (EGF-L, aa199–323) of human TN-R provided by GenBank (ID: NP 003276.3), coupled to keyhole limpet hemocyanin, and used to immunize rabbit as antigen for antibody Induction in SC contused rats	The rabbit-derived antiserum was collected after the 4th immunization and purified by antigen-coupling sepharose chromatography. And the serum before immunization was collected for control, TN-R polyclonal antibody (83 g/mL, n = 12) was locally delivered to lesion sites of rats at a rate of 2.5 L/h (±0.05 L/h) for 4 weeks	Treatment with anti-TN-R antibodies increases axonal plasticity and sprouting by attenuating the RhoA activation and improving functional recovery, therefore, specific TN-R antagonists could be a useful therapeutic approach for promoting neural repair after SCI.	You et al. (2012)
5	Adult female Sprague-Dawley (SD) rats SC	A recombinant plasmid, pcDNAGMCSF-NgR-PirB was constructed. The recombinant plasmid then formed a composite nucleic acid vaccine with liposome, and the effects of the antisera on neurite outgrowth of human neuroblastoma SH-SY5Y cells, axonal regeneration and functional recovery in spinal cord injured rats were further investigated	Five-week-old female SD rats were immunized with 100 μg of nucleic acid vaccine (formed by mixing the recombinant plasmids pcDNA-GMCSF-NgR-PirB) and liposome at equal volume and incubation for 20 min at room temperature) by injection into musculus tibialis bilaterally once weekly for 6 weeks. After the sixth immunization, rats were exposed to SCI at the T9-T11 level	This study showed that vaccination targeting the Nogo-66 receptor and paired immunoglobulin-like receptor B could stimulate the production of antibodies against NgR and PirB, blocked the inhibitory effects mediated by various MAIs, and promote nerve regeneration at the site of injury, and promote functional recovery after spinal cord injury	Lu et al. (2019)
6	SC contusion in C57BL/6 mice by laminectomy at T9–T10 level of spinal cord	Adoptive transfer of Th1-conditioned lymphocytes which trigger the axonal sprouting at the site of injury. The expression of NT-3 was higher in Th1 cells treated animals as compared to those received Th2 or Th17 cells and control	Intraperitoneal adoptive transfer of type 1 helper T (Th1)-conditioned cells (5.0 × 10^6^ or 1.0 × 10^7^) suspended in 500 mL PBS were injected into mice after 4 days of SCI.	This study suggested that Th1-conditioned cells, but not Th2- or Th17-conditioned cells, facilitated the recovery of sensorimotor function after SCI. Th1-conditioned lymphocytes secreted significantly higher levels of neurotrophic factor, neurotrophin 3 (NT-3) and promoted the recovery of locomotor activity and tactile sensation to induce the regrowth of corticospinal tract and serotonergic fibres. It was found that microglia and macrophages were activated by the transfer of Th1-conditioned cells and the M2 subtype of microglia/macrophages was upregulated	Ishii et al. (2012)
7	SC contusion model of adult female Sprague-Dawley (SPD) rats	To observe the role of Neural-Derived Peptide vaccination against apoptosis after traumatic Injury, adult rats were subjected to SC contusion and immunized either with A91 or phosphate-buffered saline (PBS; control group). Seven days after injury, animals were euthanized to evaluate the number of apoptotic cells at the injury site	Animals were injected subcutaneously at the base of the tail with 150 g of A91, immediately after injury (no longer than 60 min after injury) immunizing with this peptide reduced the apoptosis at the site of injury	The present study shows the beneficial effect of INDPs on apoptosis, where A91 immunization reduces the number of apoptotic cells immunization with A91 lowered NO and iNOS and deviates the immune response towards an anti-inflammatory phenotype; all these factors may correlate with a decrease in total TNF-concentrations and therefore result in less TNF-mediated apoptosis	Rodríguez-Barrera et al. (2013)
8	Contusive SCI model of adult rats	Adult rats were immunized with the NgR vaccine at 1 week after a contusive SCI at the thoracic level, and the NSCs, obtained from green fluorescent protein transgenic rats, were transplanted into the injury site at 8 weeks post-injury. The rats were randomly divided into seven groups i.e., sham-operated; SCI + NS (normal saline); SCI + NSCs; SCI + vaccine V); SCI + vaccine + NSCs (VC); SCI + vaccine + NSCs + NgR antibody (VCA); SCI + vaccine + NSCs + NgR antibody + methylprednisolone (MP) (VCAM)	The purified recombinant NgR protein was used as an anti-NgR vaccine and administered after 1 week of contusive SCI at the thoracic level, While NSCs were transplanted 8 weeks post-injury. Immediately after surgery, the rats in VCA and VCAM groups received a bolus injection of 50 mL NgR antibody (1:1,000, prepared by our lab) diluted in 0.5 mL NS, or injection of the NgR antibody plus 30 mg/kg MP.	The combined therapy with NgR vaccination and NSC transplantation protected more ventral horn motor neurones in the injured spinal cord and greater functional recovery than when they were used alone. Moreover, *in vivo*, NgR vaccination promoted the migration of NSCs at the site of injury and induced their differentiation into neurones and oligodendrocytes	Xu et al. (2011)
9	Contusive SCI was induced by laminectomy at the T9 level in adult BALB/c mice	Vaccination with dendritic cells pulsed with homogenate proteins in the SCI mice model to investigate the changes of neurotrophins, cytokines and T-cell at the site of SCI in mice after vaccination with hpDCs.	Three groups i.e., hpDCs, DCs (control), or PBS (control) were injected into the peritoneum (at the right mid-abdomen) of injured mouse spinal cords at an appropriate number and volume, 24 h after SCI. Functional recovery of the spinal cord was measured weekly using the Basso Mouse Scale (BMS) and confirmed by histological and immunohistochemical analysis	This study reveals that 84 days of immunization with hpDCs significantly promote SCI repair by up-regulating BDNF, NT-3, IL-4, and IFN at the injury site and the number of cysts in the hpDCs group decreased significantly compared with control groups. BDNF, NT-3, IL-4 and IFN- levels at the injured site as well as BDNF and NT-3 levels in the supernatant of cultured T-cell from the hpDCs group were significantly higher than in control groups	Wang et al. (2013)
10	Adult lewis Sprague Dawley female rats were subjected to SC contusion at the T9 level	To investigate whether the therapeutic window can accommodate two treatment modalities, i.e., i) immediate administration of MP (methylprednisolone) and ii) delayed immunization with T-cell-based vaccination and in rats	Firstly, cyclosporin (3 mg/kg) was injected immediately after the injury twice (after every 12 h interval) while sodium succinate MP (30 mg/kg) was injected into the tail vein in one or several doses after the SCI. For active immunization rats were immunized with A91 emulsified in an equal volume of Freund’s adjuvant CFA.	This study showed that if MP was injected concomitantly with the therapeutic vaccination, the beneficial effects of the vaccination were diminished. However, if MP was given immediately after the spinal injury followed by vaccination after the delay of 48 h, then the beneficial effects of MP were retained. Collectively indicating that the therapeutic window after SCI can accommodate immediate administration of MP plus a delayed therapeutic vaccination	Ibarra et al. (2004)
11	Adult female Sprague Dawley rats were subjected to moderate SC contusion	This study developed a reproducible surgical procedure to remove the glial scar followed by immunization with INDP. In the first step INDP in combination with scar removal was found to increase the number of regenerating axons at the site of injury. The second step compared whether the results of the combined therapy provided better results when compared to INDP alone	After 2 months of SCI within a 60 min frame after injury, rats were immunized subcutaneously at the base of the tail with 200 μg of A91 in phosphate-buffered saline (PBS), emulsified in an equal volume of complete Freund’s adjuvant (CFA) containing 0.5 mg/mL *Mycobacterium tuberculosis* followed by removal and inhibition of scar formation with subsequent analyses carried out over the two following month. 1) sham-operated rats (SC is exposed but scar tissue is not removed) immunized with PBS (n = 9); 2) rats with scar removal alone (n = 9); 3) rats with scar removal + INDP (n = 9)	These findings suggest that INDP, both alone and in combination with scar removal, can potentially modify the non-permissive micro-environment, i.e., an increased expression of the gene encoding for INFγ in rats treated either with scar removal + INDP or with INDP alone). This finding indicated that animals treated only with scar removal did not present a significant increase in the expression of the genes encoding for BDNF or IGF-1. However, scar removal along with INDP provides an optimized microenvironment to achieve the best conditions for neurological improvement	Rodríguez-Barrera et al. (2017)
12	Contusive SCI in adult Lewis or Sprague Dawley (SPD) rats	This study showed that post-injury injection of bone marrow-derived DCs (Dendritic cells) pulsed with encephalitogenic or non-encephalitogenic peptides derived from myelin basic protein, when administered (either systemically or locally by injection into the lesion site) up to 12 days after the injury, pronounced the recovery from severe incomplete SCI.	In this study DCs specifically pulsed with a peptide (a segment of MBP in which the amino acid lysine in position 91 is replaced by alanine), were injected into the site of spinal cord contusion in rats. This modified peptide (A91) has been shown to cross-react with the original encephalitogenic peptide, activating weak self-reacting T-cell and thereby inducing autoimmunity without the risk of inducing experimental autoimmune encephalomyelitis (EAE), thus a well-regulated primary immune response was created to provide neuroprotection	In this study use of antigen-specific DCs may represent an effective way to obtain, *via* transient induction of an autoimmune response, the maximal benefit of immune-mediated repair and maintenance as well as protection against self-destructive compounds, i.e., DC-mediated stimulated systemic T-cell-dependent immune response and diminished formation of cysts and scar tissue accompanied the improved functional recovery	Hauben and Schwartz (2003)
13	Adult Fischer 344 rats model was inflicted with moderate SC injury	SC contusion model was immunized with A91 Peptide or Copolymer-1, to observe its effects on the gene expression of inducible nitric oxide (NO) synthase produced by glial cells when co-cultured with autoreactive T-cell. This study also evaluated the expression of the inducible form of nitric oxide synthase (iNOS) at the injury site of SC-injured animals	The animal model was immunized subcutaneously at the base of the tail with 200 l g of A91, Cop-1, or 0.15 M PBS. In the first experiment. The efficacy of immunization with A91 or Cop-1 for inducing a specific immune response was evaluated., in 2nd experiment; effect of anti-A91- or antiCop-1-specific T-cell on the production of NO by glial cells and the amount of NO at the site of injury was assessed. This experiment explored the expression of the iNOS gene after immunization with A91, Cop-1, or PBS in rats (n 5 4 per group) or mice (n 5 4 per group) with SC injury	The present study provides substantial evidence on the inhibitory effect of INDP on NO production, to aid in understanding the mechanisms through which protective autoimmunity promotes neuroprotection after SCI. The neural-derived peptides A91 and Cop-1 were used to immunize rats with SC injury. *In vitro* studies showed that INDP significantly reduced the production of NO by glial cells. While *in vivo* experiments demonstrated that INDP reduced the amount of NO and iNOS gene expression at the site of injury	García et al. (2012)
14	SC contusion in the Adult Fischer 344 rat model was designed by severe and moderate injury at the T9 level of the spinal cord	In this study, the effects of INDP (two different peptides: A91 and Cop-1) on SC-contused rats were evaluated by the expression of inflammation-related genes i.e., IFNɣ, IL6, IL12, TNFα, IL-1β, IL-10, IL-4, and IGF-1	In this study, experimental animal models were subjected to either moderate or severe SC contusion and 60 min after the onset of SCI, rats were immunized subcutaneously at the base of the tail with 150 μg of A91 or Cop-1 dissolved in 0.15 M phosphate-buffered saline (PBS) (experimental groups), or only with PBS (control groups: SC injury + immunization with PBS and sham-operated animals). Both peptides and PBS alone were emulsified in an equal volume of complete Freund’s adjuvant (CFA) containing 0.5 mg/mL of *Mycobacterium tuberculosis*	After a moderate SCI, INDP reduced pro-inflammatory gene expression and generated a microenvironment prone to neuroprotection while a significant reduction in the expression of IL6, IL1β, and TNFα but an increase in IL10, IL4, and IGF-1 expression was observed. There was no effect on IL-12 and INFɣ. In contrast, the opposite pattern was observed when rats were subjected to a severe spinal cord contusion. Immunization with either peptide caused a significant increase in the expression of IL-12, IL-1β, IFNɣ (pro-inflammatory genes), and IGF-1. There was no effect on IL-4 and IL-10 compared to controls	García et al. (2018)
15	Female Sprague-Dawley rat SCI model by laminectomy at the T9-T10 thoracic Level	In this study, a clinically relevant spinal cord injury contusion model was used to investigate the effect of immunotherapy with Glatiramer acetate (GA) against Myelin basic protein (MBP) antigen in the acute phase after spinal cord injury	Sprague-Dawley (SD) rats were randomly divided into high-dose Glatiramer acetate treated for 28 consecutive days after SCI (2 mg/kg GA, high-dose) and low-dose Glatiramer acetate treated for 28 consecutive days after SCI (0.5 mg/kg GA, n ¼ 12), For analysis, high-dose GA (2 mg/kg) treatment group was compared with its low-dose (0.5 mg/kg) treatment, SCI control, and Sham control rat groups	It was observed that high-dose (2 mg/kg) administration of GA impaired locomotor recovery, which worsen lesion pathology with higher spinal neuron loss in the acute phase after spinal cord injury. Moreover, high-dose GA in the acute phase after SCI amplified autoimmunity against MBP with increasing proliferative responses. Thus suggesting that high-dosage immunotherapy with GA in the acute phase after spinal cord injury exhibited neurodestructive effects which further devastated any neuroprotective effect of T-cell	Askarifirouzjaei et al. (2019)

* MBP (myelin basic protein), SC (spinal cord), PA (protective autoimmunity), LP (lipid peroxidation), granulocyte-macrophage colony-stimulating factor (GM-CSF).

Non-etheless, preclinical studies have indicated that Nogo receptors are among the most important as well as most complex molecular candidates for immunotherapeutic strategy and each component holds a specific mode of action in autoimmunity-induced demyelination of the CNS. For instance, Nogo (623–640), “a subtype of Nogo receptor”, has been reported to possess benign immunogenic properties to intervene in axonal damage while promoting axonal regeneration, these findings suggest that inflammation of the CNS could potentially limit the efficiency of DNA vaccination by interfering axonal regeneration (Karnezis et al., 2004). Hence, inconsistent and variable results in preclinical studies and the complexity of inhibitory signalling mediated by MAIs are the major challenges that not only hampered the clinical trials of immunotherapeutic strategy but also limited the preclinical stages of immunotherapy. On the other hand, to circumvent these limitations, co-regulation of multiple targets and modification of myelin inhibitor’s antigens have shown positive outcomes in promoting functional recovery.

Though immunosuppression regimens have employed steroids such as methylprednisolone for the treatment of SCI patients which unfortunately met little or no success due to a lack of their ability to distinguish between pro- and anti-inflammatory activities of the innate immune response (Cabrera-Aldana et al., 2017; Sunshineet al, 2017). Moreover, another anti-inflammatory drug minocycline (tetracycline-class antibiotic) has been clinically applied and showed significant neuroprotective effects by inhibiting microglia activation and downregulation of TNFα, IL-1β, and Cox2 after CNS injury which ultimately suppresses the innate immune response at multiple stages after SCI injury (Chin et al., 2017; Simon et al., 2018). While Phase II Clinical trials showed positive effects of minocycline in improving motor score after SCI (Casha et al., 2012). Therefore, a Phase III clinical trial entitled ‘Minocycline in Acute Spinal Cord Injury has been approved (https://clinicaltrials.gov/ct2/show/NCT01828203) (Thibault-Halman et al, 2017). Despite promising advancement in the application of immunotherapy to CNS injury in preclinical trials, safety and delayed effects remained the major challenge for the clinical translation of these strategies (Grady et al., 2022). Therefore, the development of immunotherapeutic strategies for the clinical setting requires close monitoring and further comprehensive studies to unravel the underlying mechanism to ensure the safety and efficacy of these strategies to avoid any risk of neurological damage (Lu et al., 2016). It is also noteworthy that physiological differences between human and animal models have a significant impact on the clinical translation of preclinical trials of DNA vaccination (Cavenaugh et al., 2011). Therefore, to enhance clinical safety and to overcome these challenges that prevent the progression of immunotherapeutic strategies toward clinical trials, it is necessary to understand the key molecular events associated with the pathophysiological response of the human body to the application of these therapies (Ulmer et al., 2006; Tomchuck et al., 2012).

### 2.3 Barriers to clinical application of immunotherapy and future perspective

Although preclinical studies endorse immunotherapy as a promising therapeutic modality to promote functional recovery after SCI, yet, there is a lack of clinical evidence to support the practical application of these strategies (Rodríguez-Barrera et al., 2013). Unlike most preclinical studies which credited regulation of apoptosis and oxidative for combat the neuro-damaging effects, the pathophysiology of the human body in response to these immunotherapeutic strategies is far more complex, involving the interplay between innate and adaptive along with immune system regulation comprising a cascade of molecular events and signalling pathways in terms of axonal regeneration and neuro recovery. Therefore, more crucial aspects i.e., autoimmunity, demyelination, neuronal loss, and axonal degeneration, particularly, the risk of autoimmune diseases should be considered to treat patients with SCI. However, such studies are still at the experimental stage and there is a darn lack of clinical evidence to support the practical application of these strategies. Besides, the impermeability of the blood-spinal cord barrier (BSCB) is the major obstacle to the success of immunotherapeutic strategies, which prevents them from reaching the target lesion areas and results in poor clinical effectiveness (Shen et al., 2021). Further insight has indicated that BSCB can promote a regulatory and protective microenvironment in the cellular constituents of the spinal cord. Initially, due to a lack of knowledge about structural physiology and its function, the BSCB was regarded as a morphological extension of the BBB into the spinal cord. However, recent studies have indicated the specific morphological differences between the endothelial cells of BBB and BSCB. Thus BSCB is a relatively independent physiological and structural unit with differences in cell junction protein expression and increased permeability as compared to the BBB (Prockop et al., 1995). Moreover, the development of an efficient mode of drug administration can combat the poor permeability across the BSCB and enhance the clinical efficiency of immunotherapy. Accordingly, a recent study found that the efficiency of the non-invasive method of intranasal administration of drugs *via* the olfactory and trigeminal nerves to the brain and spinal cord can be enhanced by nanoparticles (Liu et al., 2010). Intriguingly, a novel intravenous administration of biodegradable immune-modifying nanoparticles after both moderate and severe SCI has been reported to reduce the infiltration of monocyte at the site of injury and provide an analogously safer strategy to promote functional recovery (Bharadwaj et al., 2018). Hence, overcoming the barrier of BSCB by understanding its role in drug transmission can aid in devising promising systems for targeted drug delivery towards BSCB.

Intriguingly, recent studies have suggested that the application of MSCs-derived exosomes as an important strategy to overcome the BSC barrier and induce neural recovery (Liu, Wz et al., 2021). Being significantly smaller than MSCs, MSCs-derived exosomes could easily reach the target site (Wang X et al., 2019). Importantly, it has been reported that exosomes derived from hUC-MSCs could stimulate the polarization of macrophages from the M1 phenotype to the M2 phenotype (Sun G et al., 2018). For instance, Lankford et al. have shown that intravenously injected MSC-derived exosomes could quickly reach the site of injury at the spinal cord (instead of transferring to the uninjured spinal cord) and specifically bind to M2 macrophages, indicating that M2 macrophages can alleviate SCI (Lankford et al., 2018). Moreover, Wang et al. reported that BMSC-derived exosome treatment effectively reduces SCI-induced A1 astrocytes by inhibiting nuclear translocation of NF-κB p65 (since the secondary inflammation of SCI has been reported being regulated by the NF-κB pathway (Conti A et al., 2003; Wang L et al 2018). Hence suggesting that future research must bring an exosome-based immunomodulatory strategy to devise an effective therapeutic strategy to regulate the immune response and promote neural recovery after SCI.

Another significant issue that deteriorates the quality of life in SCI patients is gastrointestinal dysfunction; however, instead of considering the impaired gut function and the role of gut microbiota, most research works remained focused on bowel management. The fact that distinct dysbiosis by alteration of gut microbiota has been indicated to aggravate secondary spinal cord injury and can be attributed to the severity of the lesion and degree of complete or incomplete injury. Thus resulting in severe comorbidities in SCI patients (Bazzocchi, G et al., 2021). Moreover, inflammatory changes in the gut are likely to contribute to reduced intestinal function and barrier integrity, particularly, a greater abundance and/or expansion of specific pathobionts after SCI has been indicated to trigger Th17 response, which aggravates inflammation (Jogia T & Ruitenberg MJ., 2020). Moreover, gut microbes have been indicated to release metabolites and signalling molecules such as short-chain fatty acids (SCFAs) that play an important role in configuring the peripheral immunomodulatory processes (Norton L., 2010). Hence, these immunomodulatory activities of gut microbes can potentially alter systemic immune function that can guide the researcher in designing an effective therapeutic modality to reduce the severe commodities faced by SCI patients.

Non-etheless, the most critical challenge is the clinically safe application of an immunotherapeutic strategy to avoid the side effects of inflammatory reactions. Aside from considering the crucial role of immunoregulation of T-cell, previous researchers have also attempted to develop anti-inflammatory drugs that can inhibit the production of pro-inflammatory cytokines and promote anti-inflammatory cytokines to treat SCI. Moreover, it is also imperative to ensure that these antibodies will not adversely affect healthy myelin and will not cause adverse immunological reactions (Ren et al., 2020). Accordingly, a clinical study has also practised the use of xenogeneic ECM bioscaffolds i.e., autologous microglia/macrophages (conditioned with immunomodulatory factors). The outcome of this study showed that autologous microglia/macrophages conditioned with immunomodulatory factors imitate the M2-like phenotype before transplantation and significantly aid in the reduction of scar formation by promoting tissue repair at the site of injury in the spinal cord (Li et al., 2017). However, despite these positive outcomes, the menace of inflammatory reaction and its deleterious effects cannot Passover. Non-etheless, in recent years replacement of cell therapy with exosomes (due to their properties of stimulating phagocytosis of microglia with minimum immunogenicity and active cellular communications along with efficient BBB permeability) has shown the advantage of overcoming the deleterious effects of inflammatory reactions (Zhang et al., 2017). Therefore, immunotherapy can be further modified by the combination of inflammasome-containing exosomes which allows smooth fusion of this therapeutic technique with peripheral immune cells and potentially promote inflammatory and immune response for cell recovery with minimum side effects (Lin et al., 2017).

For safe and efficient clinical application of immunotherapy and to avoid the limitation of short half-lives and low concentrations of therapeutic molecules at the site of injury, it is essential to determine the optimal dosage and timing (peculiarly, the time interval between repeated injections) for the next-generation of inhibitory and regulatory molecules. For instance, a previous study has reported that combined use of the steroid drug methylprednisolone (MP) with the vaccination strategy on rat models of SCI showed no beneficial outcome, however, when the timings of this strategy were modified with immediate injection of MP after the onset of injury and delayed administration of vaccination within the time window of 48 h, significantly improved outcome of vaccination. This suggests that optimizing with immediate administration of MP but a delayed therapeutic vaccination can significantly improve the outcome of therapy (Ibarra et al., 2004). A summary of studies using immunotherapy has been indicated in table 1.

Despite progressive research in preclinical trials of immunotherapeutic strategies for SCI, the clinical implication could hardly meet any significant outcome due to the failure in establishing any safety and efficacy parameters that can assure the absence of risks associated with its direct clinical application. While failure in the translation of animal trial studies into human patients can be attributed to the difference in the microenvironment of human and animal models. Therefore, a real-time professional set-up mimicking the challenges in human body reaction should be designed to provide a solid reference for compatibility with the human physiological system with beneficial clinical outcomes. Moreover, to establish an effective and safe therapeutic modality, it is important to unveil the molecular events associated with innate and adaptive immune responses after CNS injury to predict whether they will pose neurodegenerative or neuro-repairing effects. Hence, the interaction between immunosuppression, T-cell exhaustion, and immunoregulation after SCI and the underlying mechanism through which they aid in converting maladaptive inflammatory reaction to beneficial injury repair process after immunotherapy remains to be elucidated.

## 3 Conclusion

Collectively, the above-reported studies indicate that inflammation has both positive and detrimental effects on spinal cord repair and its relevant therapies in which innate and adaptive immune responses possess a vital role. However, clinical implications remained a challenging question. Therefore, by accounting for the dynamic and groundbreaking role of immunotherapeutic strategies for SCI, precise and extensive research plans are required to provide a reference for designing the future study with minimum risk of an exaggerated immune response and effectively overcome the ethical and clinical challenges due to healthcare policy regarding the clinical application of immunotherapy. To overcome the limitations and to obtain significant breakthroughs for the functional recovery and neurodegeneration after SCI, a precise and well-designed study plan (from the selection of appropriate myelin inhibitors or stem cell-derived molecules to informed, voluntary consent and model of delivery) is essentially required for the success of initial clinical trials and the development of an efficient therapeutic strategy based on the regenerative properties holds the confidence of scientists and clinicians to provide a safe and novel therapeutic avenue to the patients suffering from SCI.
